# Supplementary material for: Optimization of Different Acid-Catalyzed Pretreatments on Co-Production of Xylooligosaccharides and Glucose from Sorghum Stalk
Source: Polymers (Basel). 2022 Feb 21;14(4):830. doi: 10.3390/polym14040830 (PMC8963002; doi:10.3390/polym14040830)
Supplement: Supplementary file 1 [file polymers-14-00830-s001.zip › polymers-1554773-SI.pdf]

Optimization of Different Acid-Catalyzed Pretreatments on Co-Production of Xylooligosaccharides and Glucose from Sorghum Stalk

Xiaocui Yang [1], Xiaoliu Liu [2], Yequan Sheng [3,*], Hanzhou Yang [3], Xinshuai Xu [3], Yuheng Tao [4], Hui Wang [5] and Minglong Zhang[5,6,*]

1 Engineering Training Center, Nanjing Vocational University of Industry Technology, Nanjing, Jiangsu 210023, China;
2 College of Bioscience and Engineering, Hebei University of Economics and Business, Shijiazhuang 050061, China;
3 Jiangsu Co-Innovation Center of Efficient Processing and Utilization of Forest Resources, International Innovation Center for Forest Chemicals and Materials, College of Materials Science and Engineering, Nanjing Forestry University, Nanjing, Jiangsu 210037, China;
4 School of pharmacy&school of medicine, department of bioengineering, Changzhou University, Changzhou 213164, China;
5 College of Materials Science and Engineering, Central South University of Forestry and Technology, Changsha 410004, China;
6 Anhui Hongsen Hi-tech Forestry Co., Ltd, Bozhou 233600, China
* Correspondence: shengyequan@njfu.edu.cn (Y. Sheng); zmlong2008@126.com (M. Zhang)

Citation: Yang, X.; Liu X.; Sheng, Y; Yang H; Xu, X; Tao, Y; Zhang M. Optimization of Different Acid-Catalyzed Pretreatments on Co-Production of Xylooligosaccharides and Glucose from Sorghum Stalk. *Polymers* **2022**, *14*, 830. https://doi.org/10.3390/polym14040830

Academic Editor: Sudip Chakraborty

Received: 8 January 2022
Accepted: 18 February 2022
Published: 21 February 2022

Publisher's Note: MDPI stays neutral with regard to jurisdictional claims in published maps and institutional affiliations.

Copyright: © 2022 by the authors. Submitted for possible open access publication under the terms and conditions of the Creative Commons Attribution (CC BY) license (https://creativecommons.org/licenses/by/4.0/).

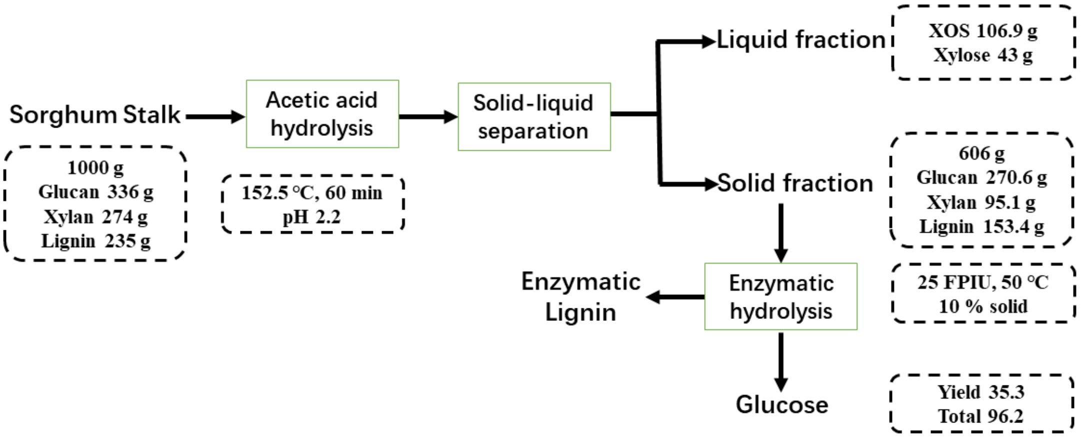

Figure S1. Mass balance of the co-production of XOS and glucose using acetic acid pretreatment from SS.

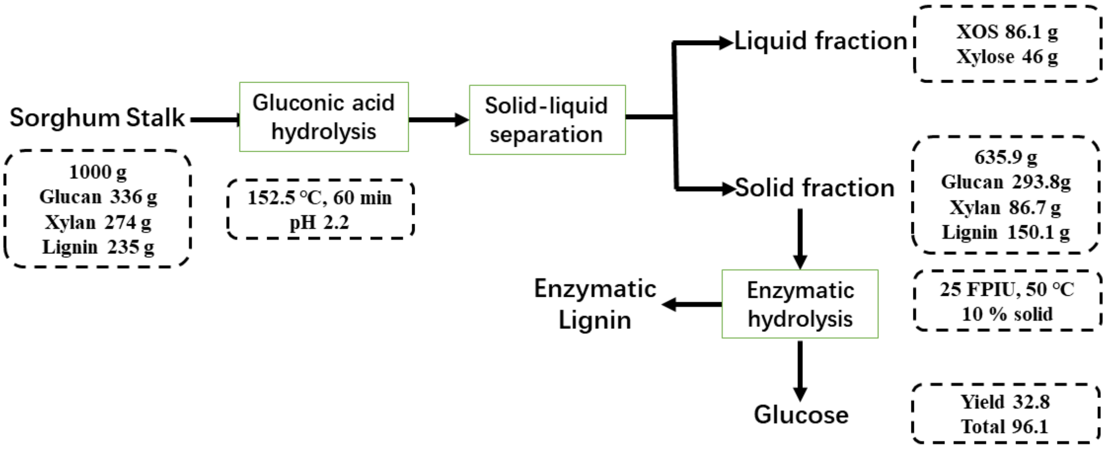

Figure S2. Mass balance of the co-production of XOS and glucose using gluconic acid pretreatment from SS.

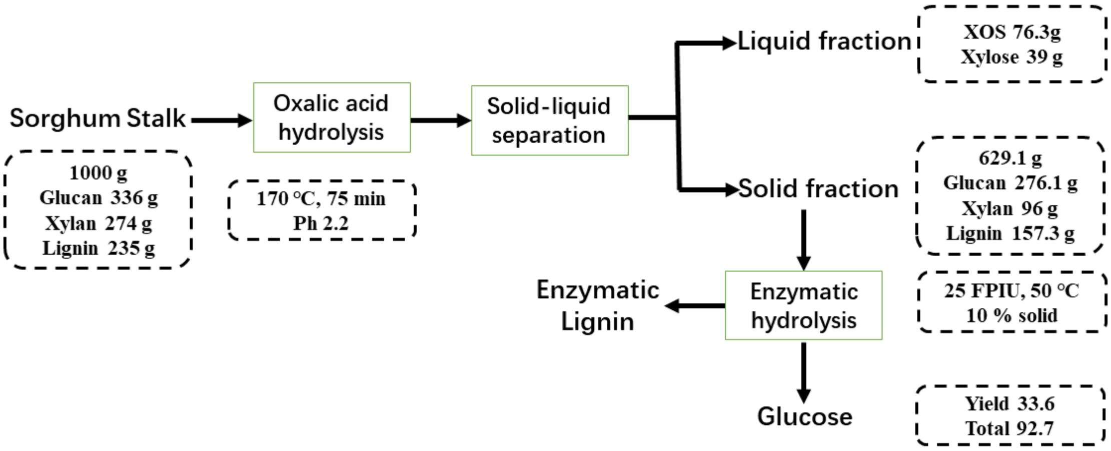

Figure S3. Mass balance of the co-production of XOS and glucose using oxalic acid pretreatment from SS.
